# High Levels of Tetrodotoxin (TTX) in Trumpet Shell *Charonia lampas* from the Portuguese Coast

**DOI:** 10.3390/toxins13040250

**Published:** 2021-03-31

**Authors:** Pedro Reis Costa, Jorge Giráldez, Susana Margarida Rodrigues, José Manuel Leão, Estefanía Pinto, Lucía Soliño, Ana Gago-Martínez

**Affiliations:** 1 IPMA—Portuguese Institute of the Sea and Atmosphere, Av. Brasília, 1449-006 Lisbon, Portugal; srodrigues@ipma.pt (S.M.R.); pereirapintoestefania@gmail.com (E.P.); lucia.solino@ipma.pt (L.S.); 2 CCMAR—Center of Marine Sciences, Campus of Gambelas, University of Algarve, 8005-139 Faro, Portugal; 3 Biomedical Research Center (CINBIO), Department of Analytical and Food Chemistry, Campus Universitario de Vigo, University of Vigo, 36310 Vigo, Spain; jgiraldez@uvigo.es (J.G.); leao@uvigo.es (J.M.L.); anagago@uvigo.es (A.G.-M.)

**Keywords:** tetrodotoxin, marine biotoxins, seafood safety, HILIC-MS/MS

## Abstract

Tetrodotoxin (TTX) is a potent neurotoxin, considered an emerging toxin in Europe where recently a safety limit of 44 µg TTX kg^−1^ was recommended by authorities. In this study, three specimens of the large gastropod trumpet shell *Charonia lampas* bought in a market in south Portugal were analyzed using a neuroblastoma cell (N2a) based assay and by LC-MS/MS. N2a toxicity was observed in the viscera of two individuals analyzed and LC-MS/MS showed very high concentrations of TTX (42.1 mg kg^−1^) and 4,9-anhydroTTX (56.3 mg kg^−1^). A third compound with *m/z* 318 and structurally related with TTX was observed. In the edible portion, i.e., the muscle, toxin levels were below the EFSA recommended limit. This study shows that trumpet shell marine snails are seafood species that may reach the markets containing low TTX levels in the edible portion but containing very high levels of TTX in non-edible portion raising concerns regarding food safety if a proper evisceration is not carried out by consumers. These results highlight the need for better understanding TTX variability in this gastropod species, which is critical to developing a proper legal framework for resources management ensuring seafood safety, and the introduction of these gastropods in the markets.

## 1. Introduction

Food-borne illnesses caused by seafood containing tetrodotoxin (TTX) are well known in East Asian countries and are primarily associated with the consumption of pufferfish and marine gastropods [1]. TTX-bearing gastropods have been extensively described, including incidents with large marine snails such as the trumpet shell (*Charonia sauliae*) [2,3,4,5]. Tetrodotoxin (TTX) is a potent neurotoxin that blocks the conduction of Na^+^ ions by binding with high specificity to voltage-gated sodium channels in mammalian muscle and nerve tissues [6,7,8]. This mode of action can lead in extreme cases to paralysis and death by respiratory and heart failure.

In Europe, there is no regulation established for TTX yet, but certain fish belonging to the family Tetraodontidae or products derived from it cannot be placed on the European markets (Regulation (EC) No. 853/2004; Regulation (EC) No. 854/2004) [9,10]. The first and only known TTX food poisoning case occurred in late 2007 when a 49 year old man consumed a trumpet shell *Charonia lampas lampas* bought in a market in South Spain. In addition to the edible portion of the snail, i.e., the white muscle of the trumpet shell, also the viscera was consumed. The symptoms began a few minutes after ingestion and were characterized by numbness in the mouth and arms, abdominal pain, nausea, vomiting, and general paralysis including the respiratory muscles [11]. TTX concentrations as high as 315 mg kg^−1^ were determined in viscera of trumpet shell and levels of 211 and 26.4 ng mL^−1^ were detected in urine and serum of the patient, respectively [12].

After this incident, the original site of collection of the causative trumpet shell was traced back and suspected to be the South coast of Portugal, where a few other specimens were caught for analysis. However, TTX was not detected in specimens caught afterwards [12]. A later survey carried out in 2009–2010, found TTX concentrations ranging only from 6.2 to 90.5 µg kg^−1^ in several gastropod species, including *C. lampas* from the North west Portuguese coast [13].

Although marine gastropods were responsible for the first TTX human intoxication in Europe, higher attention was given to TTX after its detection in bivalve mollusks. TTX levels determined in UK Pacific oysters (*Crassostrea gigas*) between 2013 and 2016, up to 253 µg kg^−1^, were much lower than levels determined in trumpet shell, but ignited the debate regarding TTX exposure in Europe, which was supported by the afterward detection of TTX in bivalve mollusks from Greece, The Netherlands, France and Italy [14,15,16,17,18].

Based on these new findings and considering TTX a marine emerging toxin the European Food Safety Authority (EFSA) has stated a scientific opinion on the risk related to the presence of TTX and TTX analogues in bivalves mollusks and marine gastropods [19]. Accordingly, concentrations lower than 44 µg TTX equiv. kg^−1^ are not expected to cause adverse effects in humans. EFSA also stated that a risk characterization for marine gastropods was not possible to carry out due to limited consumption data and lack of occurrence data.

Replying to EFSA recommendations, several studies were carried out to understand the risk posed by bivalve molluscs vectoring TTX. The first results from Portugal and Spain point to a low risk, as only trace levels or very low TTX concentrations were determined in mussels, oysters or clams [20,21,22]. TTX was also found in native Guinean puffer *Sphoeroides marmoratus* from Madeira Island, Portugal [23]. Despite these studies, little has been done to understand the occurrence of TTX in marine gastropods [24].

TTX analysis has been performed via several techniques and detection methods throughout the years [25]. While mouse bioassay has been used for decades, it is not specific to TTX and raises many ethical concerns. Alternatively, sensitive cell based assays have been developed using mouse neuroblastoma cell lines [26]. But it has been via chemical methods that detection of marine toxins has evolved in many countries including the European Union. Reverse phase liquid chromatography with tandem mass spectrometry is today well established for monitoring lipophilic toxins in shellfish in the EU and several methods have been developed and optimized for TTX detection [26,27]. However, it was after developing methods based on hydrophilic interaction chromatography (HILIC) that LC-MS/MS analysis of TTX and its analogues gained excellent resolution and sensitivity [28,29,30,31].

The aim of the present study was to evaluate the TTX toxicity in trumpet shell *Charonia lampas* from the South Portuguese coast by means of cell (N2a) based assay and HILIC-MSMS, and assess TTX distribution between edible and non edible tissues to better understand the risk for human consumption.

## 2. Results

### 2.1. Detection of TTX by Cell Based Assay

**Figure 1:**
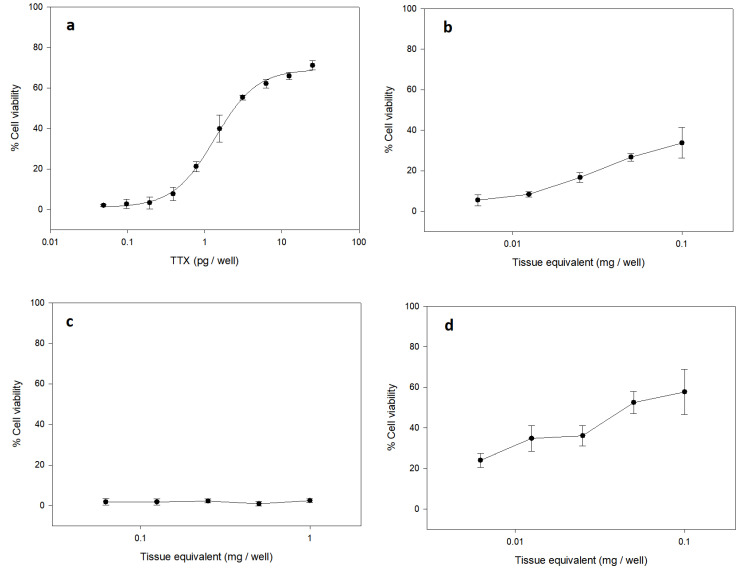
Toxic response of the cytotoxicity test for: (**a**) TTX standard with ouabain and veratridine (OV); (**b**) sample (viscera Cl-1) with toxic response (**c**) sample (muscle Cl-2) without toxic response; (**d**) sample (viscera Cl-3) with toxic response for TTX.

The assay enabled the detection of TTX based on the antagonistic effects of the toxin on a N2a cell culture exposed to sublethal concentrations of ouabain (O) and veratridine (V) (Figure 1). The noxious effects of the sodium influx triggered by O and V agonists could be inhibited by TTX. In the cell based assay, the response obtained from treatment with OV corresponds to TTX-related toxicity. TTX-like toxicity was found in the viscera of specimen 1 (Cl-1) and specimen 3 (Cl-3) (Figure 1). No toxicity was observed in the muscle of any of the samples analyzed.

### 2.2. Determination of TTX and TTX Analogues by HILIC-MSMS

After analysing the TTX toxicity by cell-based assay, samples were analyzed with HILIC-MS/MS using different MS operation modes. In order to identify the presence of TTX and possible analogues, typical ions of the TTX fragmentation pattern were screened, namely *m/z* 162.1, 178.1, 284.1, and 302.1. A common chromatographic peak at 3.2 and 4.2 min was observed for each ion fragment, suggesting the presence of TTX and analogues. These retention times were confirmed to correspond to TTX and 4,9-anhydro TTX after injecting the reference standard.

**Figure 2:**
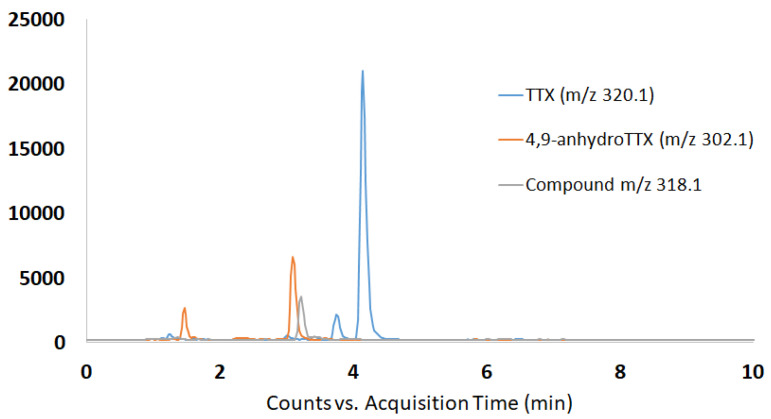
*Charonia lampas* (Cl-3, viscera). Total ion chromatograms using precursor ion mode of mass fragment ions with *m/z* 162.1 and *m/z* 178.1.

Considering that ions with *m/z* 162.1 (C_8_H_8_N_3_O) and *m/z* 178.1 (C_9_H_12_N_3_O_2_) are common for almost all TTX analogues and used as fingerprint in MS^2^ analysis [30], precursor ion search mode was then selected for monitoring parent ions that can be the source of these daughter ions. Cleaned extract samples and TTX reference standard, which contains low amount of 4,9-anhydroTTX, were used for identification purposes. Chromatographic results in terms of MS^2^ analysis, and comparison of retention times of reference standard solution and sample extract, confirmed the presence of TTX, 4,9-anhydroTTX and also a parent ion with *m/z* 318 (Figure 2).

**Figure 3:**
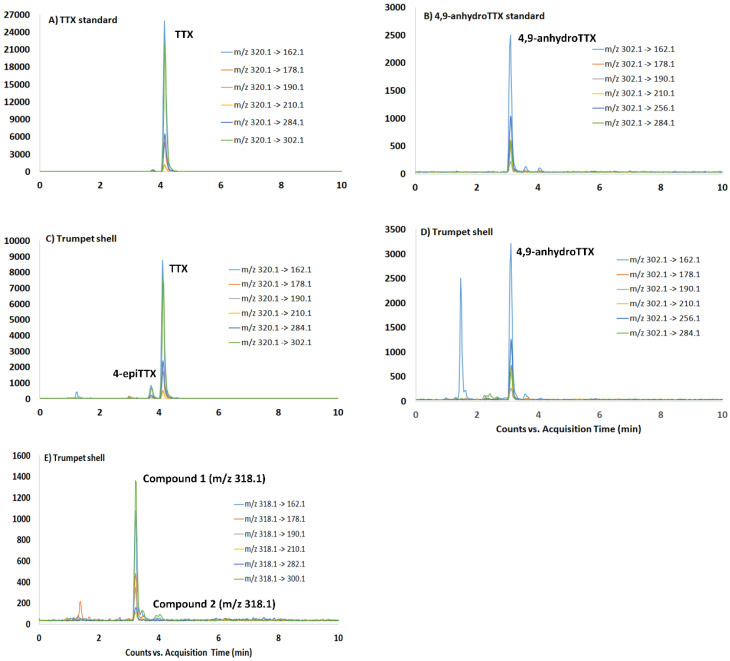
Multiple reaction monitoring (MRM) chromatograms of TTX and 4,9-anhydroTTX in certified standard (**A**,**B**), and in trumpet shell (Cl-3, viscera) sample (**C**,**D**) and, the TTX analogue with *m/z* 318 in trumpet shell (**E**).

MRM mode with six transitions including with *m/z* values 162.1; 178.1, 190.1 and 210.1 observed for TTX fragmentation was used for the identification and confirmation of the presence of TTX and additional analogues (4,9-anhydroTTX, 4-epiTTX and two compounds with *m/z* 318.1) in the trumpet shell extract (Figure 3).

**Figure 4:**
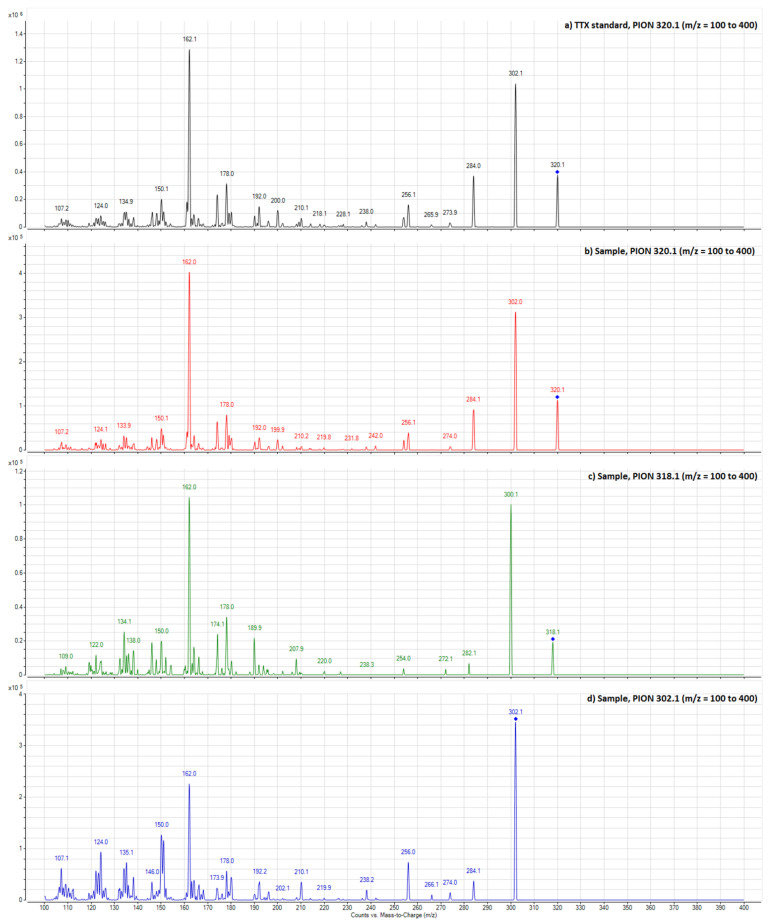
Product ion spectra of (**a**) *m/z* 320.1 of TTX standard, (**b**) *m/z* 320.1 of TTX in sample extract (Cl-3, viscera), (**c**) *m/z* 318.1 of TTX analogue in sample extract (Cl-3, viscera), and (**d**) *m/z* 302.1 of 4,9-anhydro TTX in sample extract (Cl-3, viscera).

Full scan product ion from *m*/*z* 100 to 400 was used for structural qualitative information of parent ion with *m/z* 318. For this elucidation study, TTX standard was used as reference, and similarly to TTX MS^2^, mass spectra data were obtained for 4,9-anhydroTTX and TTX analogue with *m/z* 318.1 (Figure 4).

An identical fragmentation pattern was observed for parent ions 320.1, 302.1 and 318.1 in the sample extract when compared with TTX reference standard (Figure 4). With this result, it is possible to clearly identify the presence of TTX, 4,9-anhydro TTX and the TTX analogue with *m/z* 318.1.

The analogue strictly followed the TTX fragmentation pattern in reference standard, with two water loss molecules from molecular ion and two fragments corresponding to the 2-aminohydroxyquinazolines with *m/z* 162.1 and 178.1 [27]. In addition, the MS/MS data highlighted the presence of two chromatographic peaks for trumpet shell extract, suggesting the presence of two compounds with a very similar chemical structure and the same molecular weight (Figure 3E).

Finally, the quantification of the TTX compounds present in the trumpet shell samples was performed against the TTX certified reference standard, containing certified values of TTX and 4,9-anhydroTTX. Calibration curves were prepared using matrix match standards (MMS), being 0.3 and 0.9 µg kg^−1^ the estimated values for LOD and LOQ respectively. Table 1 shows the level of TTXs toxins achieved for trumpet shell samples. Extremely high concentration of both TTX and 4,9-anhydroTTX were founded in the samples of trumpet shell viscera that provided positive results by cell-based assay. Despite the high levels determined in the viscera, the muscle tissue, which is the edible part of this seafood species showed very reduced levels, reaching 31.3 and 88.0 µg kg^−1^ of TTX and 4,9-anhydroTTX in specimen Cl-3, respectively.

## 3. Discussion

After the first human intoxication due to consumption of trumpet shell that occurred in 2007 in Spain [11], some attempts have been made to understand the accumulation of TTX in this marine organism. Rodriguez et al. [12] analyzed the trumpet shell involved in the human poisoning, determining very high levels of TTX (315 mg kg^−1^) and 5,6,11-trideoxyTTX in the viscera. However, analysis of subsequent samples collected in South Portugal did not reveal TTX contamination [12]. Further studies, carried out by Silva et al. [13,24] with the attempt to characterize the TTX occurrence in seafood species from the Portuguese coast, reported TTX below the limit of detection, or when detected it was at very low levels. These studies suggest a large variability in the occurrence of TTX in trumpet shell, which seems to vary from not detected to extremely high levels.

The present study brings more data to this topic, and once again pointing to a great variability on TTX accumulation. From the three specimens analyzed, two of them presented high TTX levels in the viscera, but in the third specimen TTX was practically absent. The trumpet shells were bought from a market in Olhão (South Portugal), without confirmation of the harvesting place, highlighting the risk for seafood safety. This gastropod species is not the target of any fisheries fleet, being most of the time a bycatch of other fisheries. It can be presumed that trumpet shells were caught in the nearby Atlantic coast of South Portugal, where this species is a delicacy offered in many regional restaurants. Nevertheless, concentrations lower than the EFSA safety limit (44 µg TTX equiv kg^−1^), were determined in the edible muscle, even though very high TTX levels were found in viscera. In addition to TTX, also the 4,9-anhydroTTX analogue and a TTX-like compound with molecular *m/z* at 318 were detected. Further studies, involving high resolution LC-MS/MS, are needed to better understand and confirm the identity of this compound. According to the available studies, the potency of TTX analogues is usually much lower than the parental compound. EFSA suggests applying a relative toxicity of 0.02 to the 4,9-anhydroTTX analogue [19].

In terms of food safety, the accumulation of TTX in the viscera of trumpet shell may resemble that of the accumulation of domoic acid in the digestive gland of scallops. In *pectinidae*, evisceration significantly reduces the toxin content enabling the adductor muscle and roe to be placed on the market. The EU Commission Decision 2002/226/EC allows harvesting scallops (*Pecten maximus* and *P. jacobaeus*) with a whole body concentration exceeding the regulatory limit for domoic acid (20 mg kg^−1^) but lower than 250 mg kg^−1^ if the parts to be placed on the market, contain less than 4.6 mg kg^−1^ [32]. After confirming the preliminary results of the present study, a similar strategy may be adopted for the trumpet shell.

Since TTX is found at high concentrations in the viscera, it can be assumed that accumulation in trumpet shell is via dietary route. Trumpet shell, *C. lampas*, is a voracious predator of starfish, although they also feed on sea urchins and holothurians [33]. Several starfish species have been associated with TTX and even TTX-producing bacteria have been isolated from starfish [2,3,34,35]. More clues on the marine trophic relationships and the toxin transfer will certainly contribute to better understand the occurrence of TTX in trumpet shell.

New toxins and new organisms acting as toxin vectors have been identified as one of the challenges to the EU harmonized monitoring programs for marine toxins [36]. In line with it, this study reports new data, which although very preliminary due to the reduced number of individuals analyzed, points out the need to improve knowledge on TTX occurrence in marine gastropods, particularly in trumpet shells. More data is needed in order to provide policymakers the relevant information for a proper management of this marine resource. As reported in this study, consumption of white muscle of trumpet shell may not represent a risk to consumers, but incorrect evisceration or consumption of the whole soft body may drastically increase food safety risk.

## 4. Materials and Methods

### 4.1. Obtention and Preparation of Samples for TTX Extraction

Three *Charonia lampas* specimens were bought in the market of Olhão (South Portugal) in late 2017. The length of the three specimens varied between 21.5 and 27.1 cm, and the weight of the eviscerated soft tissues ranged between 169.0 and 213.5 g (Table 2).

A portion of the edible muscle, located at the most anterior region of the trumpet shell, and a portion of the viscera, located at the posterior region of the animal was dissected and prepared for toxins extraction. The muscle and viscera samples were then homogenized in a blender. Toxin extraction was performed according to the Standard Operating Procedure (SOP) for determination of TTX provided by the European Union Reference Laboratory [37]. A 5 g of homogenized shellfish meat was extracted with 5 mL of 1% acetic acid by vortexing for 90 s and heating for 5 min in a boiling water bath. Samples were cooled down until room temperature was achieved and were again vortexed for another 90 s. After that, centrifugation of the samples for 10 min at 4000× *g* was conducted. A clean-up step using Graphitised Carbon SPE was carried out following the method described by [31,36,38]. A total of 5 µL of 25% _v/v_ of NH_3_ were added to 1 mL of the supernatant and was centrifuged at 10,000× *g* for 1 min before performing the SPE clean-up step.

The ENVI-Carb cartridge (Supelclean, Supelco, Sigma-Aldrich, Sintra, Portugal) was first conditioned with 3 mL of 20% _v/v_ CH_3_CN + 1% _v/v_ CH_3_COOH and 3 mL of 0.025% _v/v_ NH_3_. A 400 µL aliquot of sample extract was loaded onto the cartridge and washed with 700 µL deionized water. Toxin elution was carried out through the addition of 2 mL 20% _v/v_ CH_3_CN + 0.25% _v/v_ CH_3_COOH. The eluted extract was dried under N_2_, resuspended with culture medium and analysed by N2a assay. This same extract was diluted by transferring 100 µL to a vial and adding 300 µL of acetonitrile for LC-MSMS analysis.

### 4.2. TTX Analysis

#### 4.2.1. Reagents and Standards

Chemicals for cell-based assay: RPMI-1640 medium (R8758, Sigma-Aldrich, Irvine, UK), fetal bovine serum (F2442, Sigma-Aldrich, St. Louis, MO, USA), sodium pyruvate solution (S8636, Sigma-Aldrich, Irvine, UK), L-glutamine solution (G7513, Sigma-Aldrich, Irvine, UK), penicillin-streptomycin (P4558, Sigma-Aldrich, St. Louis, MO, USA), ouabain (O3125, Sigma-Aldrich, St. Louis, MO, USA), veratridine (V5754, Sigma-Aldrich, St. Louis, MO, USA), tetrazolium (MTT, M5655, Sigma-Aldrich, St. Louis, MO, USA).

Chemicals for LC-MSMS: acetonitrile (LC-MS grade, Merck, Darmstadt, Germany), water (LC-MS grade, J.T. Baker, Center Valley, PA, USA), ammonium hydroxide (LC-MS grade, Fluka Analytical, Steinheim, Germany), formic acid (LC-MS grade, Fluka Analytical, Steinheim, Germany), methanol (LC-MS grade) and acetic acid (LC-MS grade, Fluka Analytical, Steinheim, Germany).

Certified Tetrodotoxin (TTX) and 4,9-anhydroTTX material was purchased from CIFGA Laboratorio S.A. (Lugo, Spain) for LC-MS/MS analysis and TTX standard solution from Tocris-Bioscience (Bristol, UK) for cytotoxicity analysis.

#### 4.2.2. Cell-Based Assay (CBA)

The cell assay was performed using neuro-2a (N2a) cells purchased from the American Type Culture Collection (CCL-131, ATCC) and cultured in 75 cm^2^ culture flasks containing 25 mL RPMI-1640 medium supplemented with 10% fetal bovine serum, 1 mM sodium pyruvate solution, 2 mM L-glutamine solution, and 1000 units per litre of penicillin-streptomycin. The cell line was routinely maintained in a humidified incubator (Model 3111, Forma Scientific, Inc., Marjeta, OH, USA) at 37 °C under 5% CO_2_. The conditions used in this assay were proposed by [39,40] with slight modifications to accommodate the assay to the detection of TTX [20]. Cell cultures were incubated for approximately 24 h in 96-well culture plates. Culture wells received sample dilutions, which were prepared with RPMI-1640 medium supplemented with 1 mM sodium pyruvate, 2 mM L-glutamine, and 1000 units per litre of penicillin-streptomycin solution. Dilutions were tested in replicates of four wells. Ouabain (5 mM) and veratridine (0.5 mM) were also added and cells were incubated for 20 h. Cell viability was measured by the colorimetric method using tetrazolium metabolism. Plates were read on a spectrophotometer (Multiskan™ FC Microplate Photometer, Thermo Fisher Scientific Oy, Ratastie, Finland) at 570 nm for testing and 630 nm for reference.

#### 4.2.3. HILIC-MS/MS Analysis

HILIC-MS/MS analyses were carried out following the conditions described by Leao et al. 2018, following these summarized conditions, chromatographic separation was carried out using an Agilent 1290 Infinity LC system (Waldbronn, Germany). Chromatographic conditions used in the analysis of TTX followed the conditions described in [17]. TTX and analogues were separated injecting 2 µL of standard solution or cleaned extract on a HILIC column (Acquity UPLC Glycan BEH amide column 130A, 1.7 µm, 2.1 mm × 150 mm) from Waters (Dublin, Ireland) thermostatized at 60 °C. The samples and standard solutions in the autosampler were cooled to 4 °C. Mobile phase A was water containing 75 µL of formic acid and 300 µL of 25% _v/v_ ammonium hydroxide and mobile phase B was acetonitrile:water (7:3) _v/v_ that contains 100 µL of formic acid.

MS detection was performed using an Agilent 6495 Triple Quad MS/MS (QQQ) equipped with an iFunnel Jet Stream ESI source (Waldbronn, Germany) following the conditions described in [17]. LC-MS/MS in multiple reaction monitoring (MRM) mode was used for confirmation and quantitation purposes (Table 3). The system was calibrated with TTX standard solutions prepared in matrix match (uncontaminated shellfish cleaned extract). A five-point calibration curve of TTX with a correlation >0.990 was set up for quantification purposes and limits of detection (LOD) and quantification (LOQ) were evaluated based on the signal to noise ratios for TTX in shellfish extract with external standard addition.

Mass scan (range from 100 to 400), Precursor Ion and Product Ion search modes were used to monitoring fragment ions with *m*/*z* 162.1; 178.1 and 210.1 in order to identify and confirm the presence of TTX and possible analogues in cleaned extract samples from trumpet shell.

## Figures and Tables

**Table 1:** Concentration (µg kg^−1^) of tetrodotoxin (TTX) and its analogue 4,9-anhydroTTX determined in muscle and viscera of *Charonia lampas* by HILIC-MS/MS.

Sample	Toxins Concentration (µg kg^−1^)	
	TTX	4,9-anhydroTTX
**Edible muscle**		
Cl-1	15.0	35.4
Cl-2	<LOD	<LOD
Cl-3	31.3	88.0
**Non edible viscera**		
Cl-1	10,961.4	12,652.0
Cl-2	8.5	<LOD
Cl-3	42,163.0	56,325.5

LOD: Limit of Detection.

**Table 2:** Trumpet shell *Charonia lampas.* Shell length and weight of soft tissue.

*Charonia lampas* (Cl)	Length (cm)	Weight of Soft Tissue (g)
Cl-1	21.5	169.0
Cl-2	27.1	213.2
Cl-3	25.2	191.3

**Table 3:** MS/MS data used for the MRM acquisition of the analysis of TTX and its analogues on a 6495 Agilent Technologies mass spectrometer.

Compound	Precursor Ion	Product Ions	
TTX and 4-epi TTX	320.1	302.1	162.1
11-deoxyTTX and 5-deoxy TTX	304.1	286.1	162.1
4,9-anhydro TTX	302.1	284.1	162.1
6, 11-dideoxy TTX	288.1	270.1	162.1
5,6,11- trideoxy TTX	272.1	254.1	162.1
